# Special Issue “Precision Oncology in Melanoma Progression”

**DOI:** 10.3390/ijms22147723

**Published:** 2021-07-20

**Authors:** Simona D’Aguanno

**Affiliations:** Preclinical Models and New Therapeutic Agents Unit, IRCCS Regina Elena National Cancer Institute, Via Elio Chianesi 53, 00144 Rome, Italy; simona.daguanno@ifo.gov.it; Tel.: +39-0652-666-891

Melanoma represents the most malignant type of skin cancer, with increasing incidence worldwide. Melanoma malignancy originates from pigment-producing melanocytes in the skin (cutaneous melanoma), in the choroid, ciliary body and iris of the eye (uveal melanoma) or in mucosal membranes from different sites (mucosal melanoma) [1]. Cutaneous melanoma (here referred simply as melanoma) is the most studied subtype among the three. Different predisposing factors to melanoma development have been reported, such as exposure to ultraviolet light radiation from sunlight, congenital and acquired melanocytic nevi, genetic susceptibility and family history [1]. As concerning somatic genetic alteration associated to melanoma, 40–50% of all melanoma patients harbor an activating BRAF mutation (mostly BRAF V600E), 20–30% NRAS mutations, 10–15% NF1 mutations and 1–3% KIT mutations [2]. Melanoma is characterized by early lymphatic dissemination in the regional lymph nodes and by lymph node metastases that significantly influence the staging, prognosis and clinical approach [3]. Melanoma classification based on Tumor-Node-Metastasis (TNM) staging is represented by stage I-II (patients with local disease), stage III (node-positive disease) and stage IV (advanced or metastatic disease). Most of the patients affected by melanoma are successfully treated with surgical excision of the lesion, when early diagnosed [2]. Unresectable or metastatic melanoma patients are treated with MAPK molecular targeted therapy directed at oncogenic BRAF (vemurafenib and dabrafenib) alone or in combination with MEK inhibitor (cobimetinib and trametinib). Improved life expectancy has been also achieved with the development of immune checkpoint blockade strategies targeting the programmed death receptor-1 (PD-1) or the cytotoxic T-lymphocyte-associated antigen 4 (CTLA-4) [2]. Even though the introduction of BRAF and MEK inhibitors and immunotherapy has drastically improved the prognosis of melanoma patients [4], therapeutic resistance eventually ensues, resulting in disease progression and death. Moreover, therapeutic options are still limited for patients without BRAF mutations or in relapse from current treatments. Thus, the urgent need to identify predictive and prognostic molecules and to find novel druggable targets for additional strategy of melanoma treatment, which are aspects investigated by the precision medicine.

This Special Issue comprises five review articles and four original studies on precision oncology in melanoma progression.

In the review by Ottaviano et al. [5], the authors summarized the current knowledge about BRAF biology, underling how to deep insight into BRAF gene biology is fundamental to understand both the acquired resistance mechanisms and the molecular pathways that are now being investigated in preclinical and clinical studies with the aim of improving outcomes in BRAF-mutant patients. Epidemiology and clinic-pathological correlations between BRAF mutations in melanoma have been discussed. Moreover, the modern scenario of diagnostic and laboratory-developed tests for BRAF mutational assessment has been described, including immunochemistry, Sanger sequencing, pyrosequencing, real-time PCR and next-generation sequencing (NGS). Pharmacological treatment of unresectable/metastatic BRAF mutated melanoma were reported: dabrafenib plus trametinib, vemurafenib plus cobimetinib and encorafenib plus binimetinib. Three immunocheckpoint inhibitors (ipilimumab, nivolumab, pembrolizumab) also approved alone or in combination in this setting of patients have been discussed. Finally, the authors reported results obtained in preclinical and clinical experiments regarding the possibility to combine target therapy and immunotherapy with inhibitors of cell cycle, angiogenesis and discussed the relevance of epigenetic mechanisms and tumor mutational burden, a measurement of the genetic instability in the response to therapy. A melanoma distinguishing features is the exhibition of one of the highest numbers of clones within tumors respect to all types of neoplasms [6]. Tumor heterogeneity has relevant clinical implications, being associated with worsened prognosis and being among the cause of resistance to cancer therapies [7]. In this regard, Grzywa and colleagues reported an experimental work, where they characterized the somatic variations among high and low proliferating compartments of melanoma [8]. High and low proliferating compartments from four melanoma tumors, identified by different positivity to Ki-67 antibody, were dissected using laser-capture microdissection and subjected to NGS analysis, detecting 206 variants in 42 genes in melanoma samples and finding significant differences in mutational profiles between high and low proliferation compartments.

Resistance to immunotherapy in melanoma is the topic of the review proposed by Chacon and colleagues [9]. After describing the current immunotherapy strategies in melanoma and the proposed mechanisms of resistance to immune checkpoint inhibition, the authors introduced the class of “small molecules” (SMs), consisting of a variety of novel and existing molecules developed or utilized for the purpose of targeting precise extra- and intra-cellular proteins, many of which are critical to biological processes such as cancer cell growth and replication [9]. SMs include BRAF/MEK inhibitors, oncogenic driver inhibitors and kinase inhibitors, anti-angiogenic molecules, epigenetic modifiers, dual immunomodulation molecules and novel checkpoint inhibitors (including antibodies against the checkpoint molecules LAG-3, TIM-3 and TIGIT, among others). Alternative SMs are fructose or other small carbohydrates, lipid-based therapies including short chain fatty acid derivatives and other drugs with alternative targets such as metformin (and analogues) and non-steroidal anti-inflammatory drugs (NSAIDs). The final aim of this review was to highlight the potential synergy between well-studied SM classes and immune-check point inhibitors for the treatment of metastatic melanoma, in order to overcome intrinsic or acquired mechanism of resistance. The experimental work by Kim and colleagues well fits in the context of the development of new small molecules for the treatment of melanoma [10]. In this paper, a novel GNF-7 (a type-II multi-targeted kinase inhibitor) derivative, SIJ1777, has been shown to have antiproliferative effects toward both class I (i.e., BRAF V600 mutant), sensitive to vemurafenib and class II and class III (non-V600 BRAF mutant), resistant to vemurafenib and PLX8394, melanoma cell lines. In particular, SIJ1777 inhibited the activation of MEK, ERK and AKT, induced apoptosis and significantly blocked migration, invasion and anchorage-independent growth of melanoma cells harboring BRAF class I/II/II mutations, while both vemurafenib and PLX8394 have little to no effects on melanoma cells expressing BRAF class II/III mutations [10].

The era of precision medicine has an indissoluble link with the omics sciences. Valenti and colleagues [11] summarized the state-of-the-art in different omics disciplines applied to the monitoring of the immunotherapy response in melanoma patients, focusing on genomics, transcriptomics, proteomics, metabolomics and radiomics approaches. In addition, the authors underlined the great potential of non-invasive diagnosis represented by liquid biopsy, a new prognostic and predictive technique to monitor treatment response. In this content, they described advantages and limitations in the use of circulating tumor cells, CTCs, released by primary tumor or metastasis and present in peripheral blood, ctDNAs, small fragments of nucleic acid, released by CTCs through unclear mechanisms and exosomes, vesicles surrounded by plasma membrane and released by cells into microenvironment in the management of metastatic melanoma patients [11]. In line with this review, Tonella and colleagues reiterate the urgent need of predictive and prognostic markers to improve patient management in advanced melanoma [12]. The authors summarized the main biomarkers for stage III melanoma. In addition to biomarkers identified by gene expression, CTCs and ctDNAs analysis, they reported biomarkers identified by miRNAs, methylation and protein expression analysis. Among the factors affecting therapy effectiveness, tumor microenvironment components, such as keratinocytes, cancer-associated fibroblasts (CAFs), adipocytes and immune cells, as well as components of the extracellular matrix, play a relevant role. In the review by Mazurkiewicz and colleagues [13] the authors summarized the current knowledge concerning the influence of cancer-associated cells (keratinocytes, CAFs, adipocytes) on melanomagenesis, tumor progression, invasiveness and the emergence of drug resistance in melanoma. Soluble factors and intracellular pathways involved in cross-talk between microenvironment and cancer cells are described in details, highlighting among them possible targets to improve the development of effective antitumor therapeutic strategies. Papaccio and colleagues [14] in their experimental work proposed to better characterize melanoma-associated fibroblasts. They firstly analyzed low-passage primary CAFs derived from advanced-stage primary skin melanomas, evaluating mRNA levels of a panel of markers commonly used to identify cancer-associated fibroblasts. From a functional point of view, they found that melanoma cells maintained in CAFs- supernatant showed increased in vitro migratory ability respect to those maintained in normal huma fibroblast (NHF)-supernatant. Moreover, they demonstrated that CAF-secreted factors protect melanoma cells from acute toxicity of chemotherapy, while melanoma cells influence the paracrine activity of CAFs [14]. Finally, Pawlikowska and colleagues investigated in vitro the cross-talk between melanoma and immune cells [15]. By using a human melanoma cell line in which the level of pigmentation can be controlled by the L-tyrosine concentration in culture medium, the authors investigated the effect of the suppression of melanogenesis on the melanoma cell response to Coriolus versicolor (CV) Chinese fungus extract. They found that CV- extract can induce RIPK1/RIPK3/MLKL-mediated necroptosis in depigmented melanoma cells. Interestingly, using the co-culture system, they showed that pharmacological inhibition of melanogenesis obtained by inhibition of the tyrosinase activity in melanoma cells modulates cytokine expression in co-cultured mononuclear cells. In particular, the upregulation of IL-1, IL-2, IL-6 and IL-12 mRNA expression was significant in mononuclear cells co-cultured with melanoma cells depigmented by tyrosinase inhibitors treatment, compared to pigmented cells. These results suggest that melanogenesis inhibits the reactivity of immune cells, which may influence response to therapy.

## References

[1] D’Aguanno S., Mallone F., Marenco M., Del Bufalo D., Moramarco A. (2021). Hypoxia-dependent drivers of melanoma progression. J. Exp. Clin. Cancer Res..

[2] Jenkins R.W., Fisher D.E. (2021). Treatment of Advanced Melanoma in 2020 and Beyond. J. Invest. Dermatol..

[3] Raica M., Jitariu A.A., Cimpean A.M. (2016). Lymphangiogenesis and Anti-lymphangiogenesis in Cutaneous Melanoma. Anticancer Res..

[4] Seth R., Messersmith H., Kaur V., Kirkwood J.M., Kudchadkar R., MaQuade J.L., Provenzano A., Swami U., Weber J., Alluri K. (2020). Systemic Therapy for Melanoma: ASCO Guideline. J. Clin. Oncol..

[5] Ottaviano M., Giunta E.F., Tortora M., Curvietto M., Attademo L., Bosso D., Cardalesi C., Rosanova M., Placido P.D., Pietroluongo E. (2021). BRAF Gene and Melanoma: Back to the Future. Int. J. Mol. Sci..

[6] Andor N., Graham T.A., Jansen M., Xia L.C., Aktipis C.A., Petritsch C., Ji H.P., Maley C.C. (2016). Pan-cancer analysis of the extent and consequences of intratumor heterogeneity. Nat. Med..

[7] Dagogo-Jack I., Shaw A.T. (2018). Tumour heterogeneity and resistance to cancer therapies. Nat. Rev. Clin. Oncol..

[8] Grzywa T.M., Koppolu A.A., Paskal W., Klicka K., Rydzanicz M., Wejman J., Płoski R., Włodarski P.K. (2021). Higher Mutation Burden in High Proliferation Compartments of Heterogeneous Melanoma Tumors. Int. J. Mol. Sci..

[9] Chacon A.C., Melucci A.D., Qin S.S., Prieto P.A. (2021). Thinking Small: Small Molecules as Potential Synergistic Adjuncts to Checkpoint Inhibition in Melanoma. Int. J. Mol. Sci..

[10] Kim N., Shin I., Lee J., Jeon E., Kim Y., Ryu S., Ju E., Cho W., Sim T. (2021). Novel and Potent Small Molecules against Melanoma Harboring BRAF Class I/II/III Mutants for Overcoming Drug Resistance. Int. J. Mol. Sci..

[11] Valenti F., Falcone I., Ungania S., Desiderio F., Giacomini P., Bazzichetto C., Conciatori F., Gallo E., Cognetti F., Ciliberto G. (2021). Precision Medicine and Melanoma: Multi-Omics Approaches to Monitoring the Immunotherapy Response. Int. J. Mol. Sci..

[12] Tonella L., Pala V., Ponti R., Rubatto M., Gallo G., Mastorino L., Avallone G., Merli M., Agostini A., Fava P. (2021). Prognostic and Predictive Biomarkers in Stage III Melanoma: Current Insights and Clinical Implications. Int. J. Mol. Sci..

[13] Mazurkiewicz J., Simiczyjew A., Dratkiewicz E., Ziętek M., Matkowski R., Nowak D. (2021). Stromal Cells Present in the Melanoma Niche Affect Tumor Invasiveness and Its Resistance to Therapy. Int. J. Mol. Sci..

[14] Papaccio F., Kovacs D., Bellei B., Caputo S., Migliano E., Cota C., Picardo M. (2021). Profiling Cancer-Associated Fibroblasts in Melanoma. Int. J. Mol. Sci..

[15] Pawlikowska M., Jędrzejewski T., Slominski A.T., Brożyna A.A., Wrotek S. (2021). Pigmentation Levels Affect Melanoma Responses to Coriolus versicolor Extract and Play a Crucial Role in Melanoma-Mononuclear Cell Crosstalk. Int. J. Mol. Sci..

